# Pb^2+^ Ion Sensors Employing Gold Etching Process: Comparative Investigation on Au Nanorods and Au Nanotriangles

**DOI:** 10.3390/s24020497

**Published:** 2024-01-13

**Authors:** Eun Jin Park, Tai Hwan Ha

**Affiliations:** 1 Core Research Facility and Analysis Center, Korea Research Institute of Bioscience and Biotechnology (KRIBB), 125 Gwahak-ro, Yuseong-gu, Daejeon 34141, Republic of Korea; eunjin5@kribb.re.kr; 2 Department of Nanobiotechnology, KRIBB School of Biotechnology, Korea National University of Science and Technology (UST), 217 Gajeong-ro, Yuseong-gu, Daejeon 34113, Republic of Korea

**Keywords:** Au nanomaterials, Au nanorods, Au nanotriangles, gold leaching

## Abstract

The leaching phenomenon of gold (Au) nanomaterials by Pb^2+^ ions in the presence of 2-mercaptoethanol (2-ME) and thiosulfate (S_2_O_3_^2−^ ion) has been systematically applied to a Pb^2+^ ion sensor. To further investigate the role of Pb^2+^ ions in sensors containing Au nanomaterials, we revisited the leaching conditions for Au nanorods and compared them with the results for Au nanotriangles. By monitoring the etching rate, it was revealed that Pb^2+^ ions were important for the acceleration of the etching rate mainly driven by 2-ME and S_2_O_3_^2−^ pairs, and nanomolar detection of Pb^2+^ ions were shown to be promoted through this catalytic effect. Using the etchant, the overall size of the Au nanorods decreased but showed an unusual red-shift in UV-Vis spectrum indicating increase of aspect ratio. Indeed, the length of Au nanorods decreased by 9.4% with the width decreasing by 17.4% over a 30-min reaction time. On the other hand, the Au nanotriangles with both flat sides surrounded mostly by dense Au{111} planes showed ordinary blue-shift in UV-Vis spectrum as the length of one side was reduced by 21.3%. By observing the changes in the two types of Au nanomaterials, we inferred that there was facet-dependent alloy formation with lead, and this difference resulted in Au nanotriangles showing good sensitivity, but lower detection limits compared to the Au nanorods.

## 1. Introduction

Lead, one of the heavy metals, can cause persistent and deleterious effects to human health and must be strictly controlled according to guidelines of World Health Organization (WHO) [1]; the maximum contamination level (MCL) for lead in drinking water is defined by the U.S. Environmental Protection Agency (EPA) to be 72 nM [2]. Adults with lead poisoning may exhibit symptoms such as insomnia, memory and concentration problems, infertility, kidney damage, and high blood pressure [3]. This problem is made worse to children because they are more sensitive to lead, and exposure to even low levels can cause serious and permanent damage and impair normal brain development, leading to neurodevelopmental defects [4].

Common methods for detecting Pb^2+^ ions in aquatic environmental samples include atomic absorption/emission spectrometry (AAS/AES) [5,6], inductively coupled plasma mass spectrometry (ICP-MS) [7,8], X-ray fluorescence spectrometry (XRF) [9,10], and anodic stripping voltammetry (ASV) [11]. However, those instrument-intensive methods only measure total metal ion content and often require extensive sample preparation. To overcome these shortcomings and measure lead ions more accurately, new measurement techniques are being developed: noble metal nanotechnology [12,13,14], microfluidic technology [15,16,17], spectrometry [18,19], fluorescent molecular probe [20,21], and electrochemistry [22]. Among them, optical detection can be an effective alternative due to the convenience and simplicity of detection principles, thus considerable efforts have been devoted to the development of fluorescent and colorimetric sensors [23]. The fluorescent and colorimetric sensors are divided into several categories based on Pb^2+^-sensing materials such as small molecule [24,25,26,27], calixarene [28,29,30], polymer [31,32], functional DNAzymes [33,34,35,36,37] and metallic nanoparticles [38,39,40,41,42].

One of the metallic nanoparticles, gold (Au) nanomaterials have attracted attention of many researchers. They are considered an interesting signal source for reasons that their photostability, simple transducing principle without sophisticated light source, and versatile embedding protocols in various matrices (polymeric or inorganic) for advanced studies. Therefore, they have already widely applied in many fields such as optical sensors [43,44], biomedical materials [45,46], electronics and even agriculture [47] and food technology [48]. All these applications are closely related to their chemical inertness (noble metals) and morphological diversity, which greatly influence their optoelectronics properties [49,50,51]. However, Au nanomaterials are not that inert in the nanometer regime due to their high surface energy. As the size of Au nanomaterials decreases, the excitation of localized surface plasmon resonance (LSPR), a signaling pathway, has emerged [52,53]. As a selective Pb^2+^ ion sensor, Pb^2+^-associated Au etching process have been spotlighted as a promising approach [54,55], since it could be monitored with a typical LSPR band using the naked eye and UV-Vis spectra encompassing the near-infrared (NIR) region [56]; closely related papers are listed on Table S2 in Supplementary. Hitherto, H_2_O_2_, thiourea, Hg(II), heavy metal ions (Fe(III), Cu(II), Cr(VI), Pb(II), As(III)), nitrite, and persulfate ions have been detected through morphological changes; optical sensors for corresponding heavy metal ions were demonstrated through changes of LSPR band [57]. Besides, thiol groups for immobilizing many functional ligands and halide ions in solution have shown etching properties for Au nanomaterials [58].

In this study, Au nanomaterials (nanorods and nanotriangles) were employed to detect Pb^2+^ ions in the nanomolar range with the aid of 2-mercaptoethanol (2-ME)/thiosulfate (S_2_O_3_^2−^). For this comparative experiment, we resorted to seed-mediated growth and synthesized two anisotropic Au nanomaterials under cetyltrimethyl-ammonium (CTA+) micelle system: Au nanorods (length: ~104 nm) and Au nanotriangles (edge length: ~73 nm). Au nanorods and Au nanotriangles were diversified by the amount of halide ions present during the synthesis condition of CTAB (cetyltrimethylammonium bromide) or CTAC (cetyltrimethylammonium chloride) micelles [59]. The former is fascinating due to the strict confinement of electrons and/or surface plasmon in 1D direction, while the latter features large atomically flat surfaces, providing ultrathin nanotemplates for further planar molecular arrangements. During the course of manufacturing two Au nanomaterials, the anisotropic growth by seed mediated growth results in typical LSPR bands in the NIR region. In addition, NIR nanomaterials have unique advantages to have longer a penetrating depth toward human skin that might be helpful in future applications like transdermal detection. In relatively mild etching conditions, those two Au nanomaterials showed interesting but distinct etching responses, and we reasoned our observations.

## 2. Materials and Methods

### 2.1. Chemicals

Gold(III) chloride trihydrate (HAuCl_4_∙3H_2_O, 99.9%), trisodium citrate, sodium borohydride (NaBH_4_, 99%), hexadecyltrimethyl ammonium chloride (CTAC, 25 wt% solution in water), hexadecyltrimethyl ammonium bromide (CTAB, 99%), potassium bromide (KBr, 99%), potassium iodide (KI, 99%), L-ascorbic acid (99%), lead(II) chloride (PbCl_2_, 99.9%), and sodium thiosulfate (Na_2_S_2_O_3_, 99%) were purchased from Sigma-Aldrich (St. Louis, MO, USA). 2-Mercapto ethanol was obtained from Merck (Darmstadt, Germany). Deionized water was used throughout whole experiments.

### 2.2. Preparation of Gold Seeds

A 20 mL volume of aqueous solution containing 0.25 mM HAuCl_4_ and 0.25 mM trisodium citrate was prepared in a glass vial. With vigorous stirring, 0.6 mL of ice-cold 0.1 M NaBH_4_ was quickly added to the prepared solution. After adding NaBH_4_, the solution immediately became bright pink, and the solution was maintained for 2 min with vigorous stirring. The seed solution was used after stabilization for 2 h.

### 2.3. Growth of Au Nanorods from the Seed Solution

For Au nanorods growth, a 10 mL solution including 0.25 mM HAuCl_4_ and 0.07 M CTAC was mixed with 0.1 mL KBr (70 mM) in a clean vial and then mixed with 0.1 mL of freshly prepared ascorbic acid solution (0.1 M), which resulting in a colorless solution. To this solution, 20 μL of seed solution was added and stirred thoroughly and gently for 2 h at ambient condition; UV-Vis spectrum of as grown Au nanorods is shown in Figure S1a. From the transmission electron micrograph, the average Au nanorod size (520 counts) measured was 103.5 ± 3.2 nm of length and 21.8 ± 5.3 nm of width (see Note S1 in the Supplementary) [59,60].

### 2.4. Growth of Au Nanotriangles from the Seed Solution

For the synthesis of Au nanotriangles, 0.1 M CTAB is used for the growth solution instead of CTAC. The 10 mL of growth solution containing 0.20 mM HAuCl_4_ and 20 μM KI. With CTAB, HAuCl_4_ becomes deep yellow. For making nanotriangles, the concentration of ascorbic acid was doubled to optimize production yield. After the growth solution became colorless, 30 μL of seed solution was added and gently shaken for 2 h at ambient condition; UV-Vis spectrum of as grown Au nanotriangles is shown in Figure S1b. The average edge length of Au nanotriangle (484 counts) measured from the transmission electron micrograph was 73.7 ± 8.5 nm (see Note S1 in the Supplementary).

### 2.5. Separation of Au Nanorods and Au Nanotriangles from Au Nanospheres

For separating Au nanorods and Au nanotriangles from the as-grown spherical nanoparticles, each solution was salted out after the synthesis was completed. As grown nanoparticle solution is salted out with 300 mM NaCl and 260 mM NaCl, respectively. Au nanorods and Au nanotriangles were centrifuged for 15 min at 1250 rpm for Au nanorods and at 1400 rpm for Au nanotriangles, and the supernatant was discarded. The resultant precipitates were dispersed and diluted in DW to the initial volume.

### 2.6. Typical Etching Process in the Presence of Pb^2+^/S_2_O_3_^2−^ Ions

Typically, a mixture of 900 μL of glycine-NaOH solution (5 mM, pH 10.0) and 200 μL of separated Au nanorods and Au nanotriangles were gently mixed and stabilized for a few minutes, before etching test. For etching Au nanorods, Na_2_S_2_O_3_ (0.8 mM or 0.2 mM) and Pb^2+^ ions (0~111.2 μM) were added to the prepared mixture at room temperature for 5 min and then 2-ME (3.1 mM or 22.1 mM) was added for 30 min. The condition of etching Au nanotriangles is 1.0 mM Na_2_S_2_O_3_, Pb^2+^ ions (0~72 μM), and 3.8 mM 2-ME as the same reaction time (5 min~30 min) and temperature were applied (25 °C). All experiments were replicated three or four times given a batch of prepared Au nanorods or nanotriangles, after checking the quality of separated Au nanomaterials by monitoring the peak absorbance values and linewidth of the LSPR band in full width of half maximum (FWHM).

### 2.7. Transmission Electron Microscopy (TEM)

Six microliters of each centrifuged sample were added, placed in the center of the TEM grid, and dried for 3 min. After absorbing excess liquid sample using filter paper, excess salt remained was briefly washed off by loading 5 µL of water on the TEM grid. Finally, the excess water was removed again with filter paper and dry for 30 min.

### 2.8. Instrumentation

UV-Vis absorption spectra of the solutions were taken with a UV Spectrophotometer (PhileKorea, Inc., Daejeon, Republic of Korea) and a DU 800 Spectrophotometer (Beckman Coulter, Fullerton, CA, USA) in a wavelength range from 400 to 1100 nm. Transmission electron microscopy (TEM) images were obtained by FEI Tecnai™ transmission electron microscope (FEI, Hillsboro, OR, USA) at 300 kV using a carbon film coated on copper grid.

## 3. Results

Substantial amount of isotropic Au nanoparticles appeared in as-grown samples (Figure S1a,b) could be removed through the salting out separation, resulting in Au nanorods and nanotriangles for further etching reaction. UV-Vis spectra of as-grown and separated Au nanomaterials are summarized in Figure S1. A shoulder band typically appeared at ~600 nm in as-grown nanotriangle sample corresponds to small and truncated nanotriangles or hexagonal plates, and could be successfully removed in separation process (Figure S1b,d).

**Figure 1:**
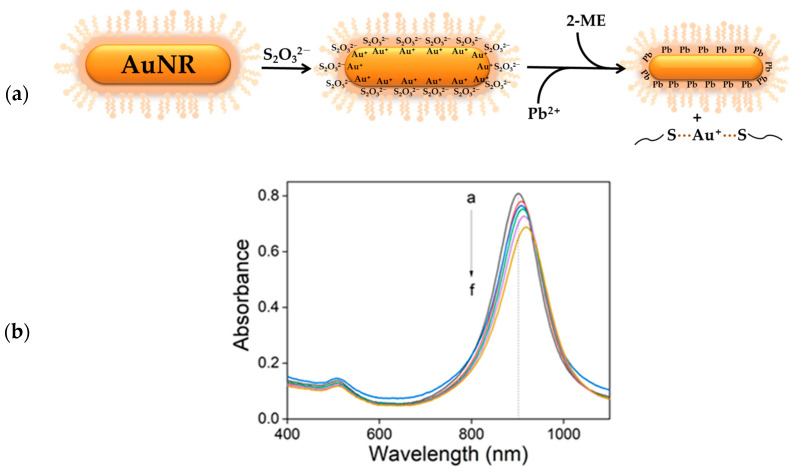
(**a**) Schematic diagram of leaching reaction of Au nanorods. (**b**) UV-Vis absorption spectra of Au nanorods on the treatment with various concentrations of PbCl_2_ (a→f: 0 μM, 1.0 μM, 2.0 μM, 4.0 μM, 8.0 μM, and 12.0 μM) at fixed concentrations of Na_2_S_2_O_3_ (0.8 mM) and 2-ME (3.0 mM) for 30 min.

Figure 1a illustrates the experimental scheme for Au nanorods adapted from the study of Huang et al. [61], who etched spherical Au nanoparticles. In our experiment, we first added a small amount of thiosulfate ions (S_2_O_3_^2−^) to the Au nanorods solution; S_2_O_3_^2−^ has a coordination capability to gold surface hindering aggregations, which could be occurred by sudden impacts on solution phase such as changes of ionic strength, solution pH, or introduction of new chemicals. If 2-ME of high concentration (>4 mM) is first introduced to reaction vessel, some nanomaterials were aggregated to change the UV-Vis spectrum slightly, so we insisted on being added Na_2_S_2_O_3_ first for all concentrations of 2-ME. On the other hand, no noticeable changes were observed when either 2-ME (3.0 mM) or Pb^2+^ ions (1.0 μM) was solely added, as shown in Figure S3a, suggesting that 2-ME or Pb^2+^ ions without S_2_O_3_^2−^ ions lack the etching power for gold nanomaterials.

After a short incubation time (20 s) of thiosulfate ions, various concentrations of Pb^2+^ ions were added and incubated for 5 min, and then a fixed concentration of 2-ME was added and incubated for another 30 min before the acquisition of UV-Vis spectra; the final concentration of 2-ME and Na_2_S_2_O_3_ was adjusted to 3.0 mM and 0.8 mM, respectively. As the Pb^2+^ concentration increases, the longitudinal plasmon band intensity decreases, as expected from the previous report [61]. However, the peak wavelength unexpectedly went to higher wavelength (red-shift, Figure 1b), implicating that the aspect ratio of Au nanorods increased [56,62]. To estimate the concentration of Pb^2+^ ions, we measured intensity changes at a fixed wavelength at 902 nm, since the peak intensity moved continuously; the bandwidth of the longitudinal LSPR band slightly increased from 121 nm to 130 nm (see Table S1), indicating almost no aggregation during the reaction.

**Figure 2:**
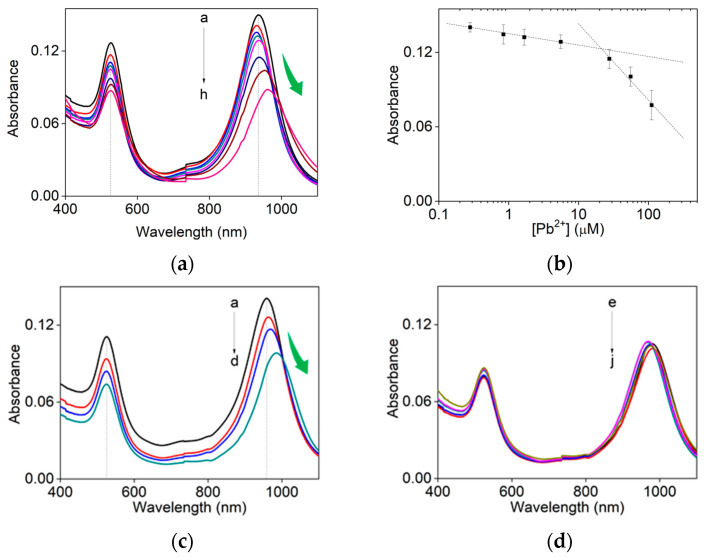
(**a**) UV-Vis absorption spectra of Au nanorods on the 30 min treatment with wider concentrations of PbCl_2_ (a→h: PbCl_2_ 0 μM, 0.3 μM, 0.8 μM, 1.7 μM, 5.6 μM, 27.6 μM, 55.6 μM and 111.2 μM) at a fixed concentration of Na_2_S_2_O_3_ (0.8 mM) and 2-ME (3.1 mM). (**b**) Scatter plotting of the absorbance values at 936 nm of (**a**). UV-Vis absorption spectra of Au nanorods at a distinct concentration set of Na_2_S_2_O_3_ (0.2 mM) and 2-ME (22.1 mM) for 30 min reaction with a concentration range of PbCl_2_ (**c**) (a→d: PbCl_2_ 0 μM, 0.11 μM, 0.33 μM, and 1.10 μM) and (**d**) (e→j: PbCl_2_ 3.3 μM, 11.0 μM, 22.1 μM, 29.4 μM, 44.1 μM and 88.2 μM).

Over a wider range of Pb^2+^ concentration, the red-shift of the longitudinal LSPR band was consistently observed as shown in Figure 2a; the repetitive experiments are summarized in the scatter plot of Figure 2b. The limit of detection (LOD) of Pb^2+^ ions for Au nanorods was estimated to be 73 nM, showing rather worse results than previous reports for spherical Au nanoparticles, with a narrow dynamic range (0.034~5.6 µM). The difference in sensitivity was manifested as shown in two slopes in Figure 2b; a higher sensitivity was measured in a range of Pb^2+^ concentration (11.2~55.6 µM) than in more diluted one. This is attributed to the substantial red-shift of peak band at higher concentration of Pb^2+^ ions, enlarging the corresponding intensity decrease.

To investigate the combined effect of Na_2_S_2_O_3_ and 2-ME, we attempted to slightly modify the etching conditions. When the 2-ME concentration was increased to 22.1 mM and the Na_2_S_2_O_3_ concentration was lowered to 0.2 mM, the Pb^2+^-dependence stopped at 3.3 µM of Pb^2+^ ion (Figure 2c); no dependence was observed at concentrations of Pb^2+^ higher than 3.3 μM, indicating that higher concentration of 2-ME overwhelm the effect of Pb^2+^ ion addition (Figure 2d); in Figure 2, as-grown Au nanorods were used for more direct comparison with spherical nanoparticles.

**Figure 3:**
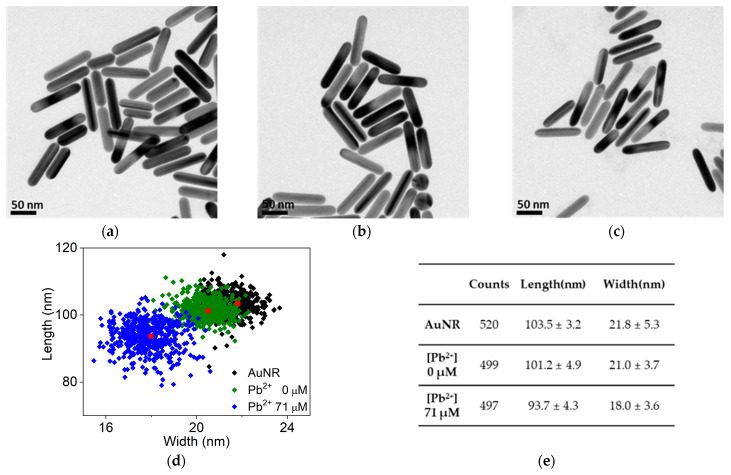
TEM images of (**a**) as grown Au nanorods, (**b**) Au nanorods treated with Na_2_S_2_O_3_ (0.9 mM), 2-ME (4.1 mM), and Pb^2+^ (0.0 μM), and (**c**) Au nanorods fully treated with Na_2_S_2_O_3_ (0.9 mM), 2-ME (4.1 mM), and Pb^2+^ (71.0 μM). (**d**) Scatter plotting of Au nanorods as the leaching reaction proceeds at different leaching conditions for 30 min (as grown AuNRs, 0 μM PbCl_2_ with Na_2_S_2_O_3_ (0.9 mM) and 2-ME (4.1 mM), and 71.0 μM PbCl_2_ with Na_2_S_2_O_3_ (0.9 mM) and 2-ME (4.1 mM)): Red dots are average values of length and width of each group of Au nanorods. (**e**) Summary table for average and standard deviation of length and width of each Au nanorod group in the scatter plot of (**d**).

Since the peak plasmon band decreases under similar etching conditions [63], we performed more detailed investigations using transmission electron microscopy (TEM), which allowed us to measure real dimension changes of Au nanorods (Figure 3a–c). At first glance, TEM observations showed little change in the size of each Au nanorod, but measurements of numerous Au nanorods (width and length) revealed that they had statistically become shorter and thinner (Figure 3a–c). As expected before, the pairing of 2-ME and Na_2_S_2_O_3_ affected the Au nanorods, shortening them by 2.3 nm and thinning them by 0.8 nm at given timescale (30 min). Further addition of Pb^2+^ ions with the same concentrations of 2-ME and Na_2_S_2_O_3_ (4.1 mM and 0.9 mM, respectively) made much more differences in both width and length; the length of Au nanorods was reduced by 9.8 nm, while the width was reduced by 3.8 nm. It is worthwhile to note that the width change (3.8 nm) is more apparent, suggesting that the aspect ratio of Au nanorods indeed increases with the addition of Pb^2+^ ions (from 4.75 to 5.21; length/width), in conjunction with the gradual decreases of their length. Furthermore, unlikely the case in the absence of Pb^2+^ ions, prolonged incubation (30 min and 1 h, Figure S2a,b) incessantly continued to etch Au nanorods; overnight incubation at the high concentration of Pb^2+^ ions obliterate all nanorods eventually. These TEM analyses confirm that the red-shift in peak wavelength observed in Figure 2a,c are indeed a reflection of the increase in the aspect ratio of the Au nanorods.

In an attempt to reveal the roles of each component of the etchant in detail, we observed the time course of spectral changes after adding each component, enabling us to obtain temporal snapshots. In Figure S3b, after initial addition of 0.8 mM Na_2_S_2_O_3_ (green) we added DW 10 μL (mimicking 0 μM Pb^2+^) and took a snapshot of spectrum and further acquired spectral snapshots at 10 min and 20 min (red). After a concentrated 2-ME was mixed with the solution, we obtained spectral snapshots (blue) with 0, 5, 10, 15, 20 min timestamp. As expected, we observed continuous decrease of the peak band at 967 nm after adding Na_2_S_2_O_3_, indicating mild etching by S_2_O_3_^2−^ ion (see Note S2 in the Supplementary). This observation is not unusual because Na_2_S_2_O_3_ has been used for gold leaching in industrial metallurgy [64]. Interestingly, the peak band blue-shifted after the addition of 2-ME, which is assumed to be etching results of 2-ME/Na_2_S_2_O_3_ without Pb^2+^; intensity decrease of 29.7% for 40 min incubation was observed with 5.6 nm blue-shift. It is also noteworthy that, 20 min after adding 2-ME, the etching reaction seems almost quenched, probably caused by formation of a protective layer such as Au_2_O_3_ in conjunction with the coordination with thiosulfate and/or 2-ME. In Figure S3c,d, we obtained similar snapshots in case of 0.9 μM and 44.1 μM of Pb^2+^ ions. Surprisingly, Pb^2+^-addition provokes red-shift of the band, while 2-ME still has a tendency to blue-shift; 3 min time interval was applied for Figure S3c,d. At Pb^2+^ concentration of 44.1 μM, the tendency of red-shift was so obvious that the initial blue-shift tendency caused by 2-ME seems to turn to the opposite direction (i.e., red-shift), shown in a snapshot at 6 min (see black arrow in Figure S4c); intensity decrease of 30.8% with a blue-shift of 9.7 nm was observed for 36 min incubation in 0.9 μM Pb^2+^ ion sample, while 46.8% intensity decrease and 4.9 nm red-shift for 44.1 μM Pb^2+^ ion sample. Enlarged version of Figure S3b–d are shown in Figure S4.

According to the control experiment in Figures S3 and S4, the major Au etchant appears to be 2-ME/Na_2_S_2_O_3_ pair under the current etching conditions. First, gold etching by only S_2_O_3_^2−^ or 2-ME/S_2_O_3_^2−^ pair have as a tendency of blue-shift, which might be caused by an isotropic (or indiscriminate) etching, thereby reducing overall size of nanorods. Second, once Pb^2+^ ion is added in the presence of S_2_O_3_^2−^ or 2-ME/S_2_O_3_^2−^, red-shift of the peak wavelength was observed, which was not the case in Au nanotriangles (see below). Third, it takes few minutes to affect the direction of the peak wavelength shift, so an incubation time of 5 min after the addition of Pb^2+^ ions should be tightly for the reproducibility of the experiments. To our best survey of literature, the red-shift of peak plasmon band, albeit small, for Au nanorods was not described before or seemed to be ignored due to minute changes the wavelength.

4Au + O_2_ + 2H_2_O + 8S_2_O_3_^2−^→4Au(S_2_O_3_)_2_^3−^ + 4OH^−^ (1)

The oxidation of gold in the presence of S_2_O_3_^2−^ ions is generally assumed to be driven by dissolved oxygen molecules (Equation (1)) and is often slowed down due to the formation of protective layers such as the form of [Au(S_2_O_3_^2−^)]^−^ [61]. Rather basic etching condition (pH 10 buffer) was chosen because p*K*_a_ of 2-ME is around (8.8~9.1) facilitating the formation of Au(2-ME)^−^ [61]. However, higher pH was avoided due to the formation of complexes like Pb(OH)_2_, PbO, Au(OH)_3_ or Au_2_O_3_ [61]. Meanwhile, chemisorbed thiolate ligands, which were usually considered forming a protective layer to preserve the morphology of Au nanomaterials and prevent uncontrolled aggregation, are also able to etch the Au surface in the presence of radicals (see Note S3 in the Supplementary) [65]. However, as mentioned earlier, when only 2-ME was added to the Au nanorod solution, there were almost no difference in the UV-Vis spectrum, suggesting that 2-ME only plays a supportive role like lowering thermodynamic energy of detached gold ions in the form of Au(2-ME)^−^ and hindering uncontrolled aggregation as a protective layer. Therefore, S_2_O_3_^2−^ ion appears to be the core component of the 2-ME/S_2_O_3_^2−^ pair, and Pb^2+^ ions act as a catalyst, which is quite reasonable in that elemental lead has a high reduction potential compared to elemental gold [66].

**Figure 4:**
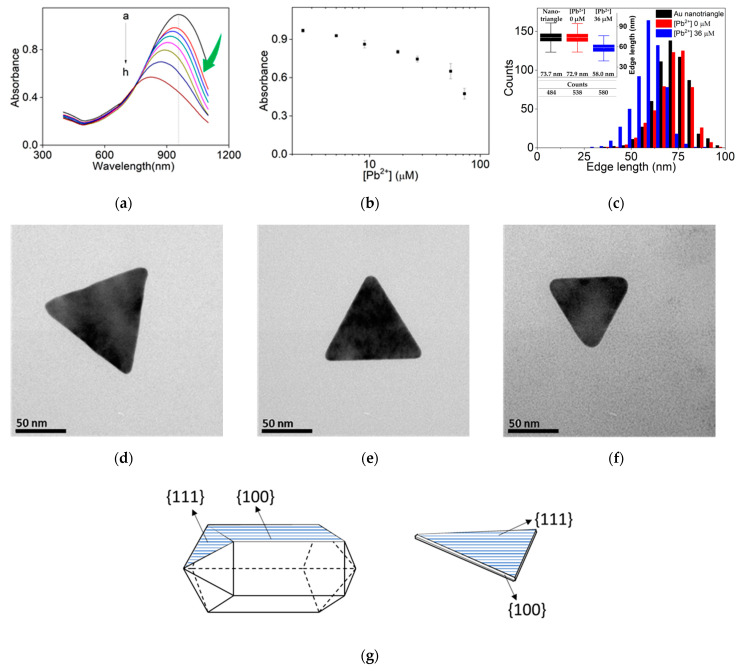
(**a**) UV-Vis absorption spectra of Au nanotriangles with different concentrations of Pb^2+^ (a→h: 0 μM, 2.5 μM, 5.0 μM, 9.0 μM, 18.0 μM, 27.0 μM, 54.0 μM, and 72.0 μM). Each group of Au nanotriangles treated by 1.0 mM of Na_2_S_2_O_3_ and 3.8 mM of 2-ME. (**b**) Dot plot of absorbance at 957 nm from UV-Vis absorption spectra of (**a**). (**c**) Distribution graph of as-grown Au nanotriangles and the etching cases. Etching was carried out under condition of 0 μM Pb^2+^ and 36.0 μM Pb^2+^ with 1.0 mM Na_2_S_2_O_3_ and 3.8 mM 2-ME. Average values are shown in the inset. Representative TEM images of single Au nanotriangles appeared in: (**d**) As grown Au nanotriangle. (**e**) Au nanotriangle treated by 1.0 mM Na_2_S_2_O_3_, 3.8 mM 2-ME and 0 μM Pb^2+^. (**f**) Au nanotriangle treated by 1.0 mM Na_2_S_2_O_3_, 3.8 mM 2-ME and 36.0 μM Pb^2+^. (**g**) Cartoons that shows the facet arrangement of typical Au nanorod and Au nanotriangle shown in this paper.

Spurred by these interesting observations, the same etching process was applied to Au nanotriangles, which is diversified by adding iodide ions [59]. As can be seen in Figure 4a, the longitudinal band located at 957 nm decreased in intensity while the peak band also shifted toward lower wavelength (blue-shift, see above), as expected from many other etching conditions. As shown in Figure 4a,b, the peak intensity at a fixed 957 nm showed a strong dependence on the concentration of Pb^2+^ ions; it is noteworthy that the steeper Pb^2+^ dependence was observed at the higher concentrations, which is a drawback for sensitive Pb^2+^ optical sensor applications. The limit of detection (LOD) of Pb^2+^ ions for Au nanotriangles was estimated to be 380 nM, showing even worse results than Au nanorods (see Note S4 in the Supplementary).

Statistics of TEM measurement for hundreds Au nanotriangles are shown in Figure 4c. As in the case of Au nanorods, the 2-ME/Na_2_S_2_O_3_ pair (3.8 mM and 1.0 mM) slightly decreased the dimension of Au nanotriangles, and Pb^2+^ ion addition showed more drastic changes in the edge length of Au nanotriangles. The original edge length (73.7 nm) decreased to 72.9 nm in the treatment of 2-ME/Na_2_S_2_O_3_, while the edge length of 58.0 nm was obtained on average with 37 μM of Pb^2+^ ion added with the etchant; red-shift of LSPR band was never observed in the nanotriangles. As shown in Figure 4d–f, the size difference appears more clearly in the TEM images. Even considering the size diversity in a collection of nanotriangles, massive size measurements revealed that the etching in the presence of Pb^2+^ ions exhibited much smaller dimension and rounded edges, confirming that the acceleration of the etching rate by Pb^2+^ addition could also apply to Au nanotriangles.

## 4. Discussion

Sequential adding of the etchant (S_2_O_3_^2−^→Pb^2+^→2-ME) prevented from any possible aggregation of nanomaterials, implicating that S_2_O_3_^2−^ ions form a protective layer on the gold surface [61]. Meanwhile, although increasing reaction temperature was reported to increase the etching rate [56], we decided to maintain the solution at room temperature (~25 °C) because more controlled and slower reaction rate was intended to reveal the differences between the two nanomaterials. We have prepared both Au nanomaterials which have excitation wavelengths in the range of NIR region (930~980 nm), which is more transparent in biological tissues than in visible range (380~700 nm) [67]. NIR suffers the least light scattering and absorption mainly caused by water molecules and heme groups in blood.

The concentrations of 2-ME and Na_2_S_2_O_3_ used in this study were relatively mild compared to the case of other reports, due to an effort to enlarge the time-window to reveal the role of each component. In our experiments, while the etching condition reduced the size of both Au nanorods and Au nanotriangles, the most intriguing and unexpected feature is the increase of the aspect ratio of Au nanorods (i.e., red-shift of the peak band ~930 nm band); detailed spectroscopic parameters such as peak wavelength and bandwidth are described in Table S1. Otherwise, the corresponding band of Au nanotriangles was observed to have a blue-shift, as reported under different etching conditions [68], which was mainly caused by rounding apexes and reduced dimensions.

**Figure 5:**
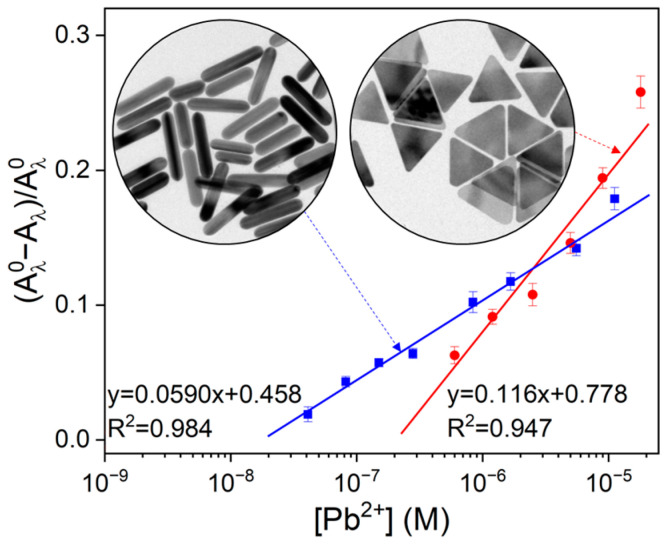
Scatter plot of normalized intensity change ((A^0^_λ_ − A_λ_)/A^0^_λ_) for Au nanorods at 936 nm and for Au nanotriangles at 957 nm. Concentration of Na_2_S_2_O_3_ (0.8 mM) and 2-ME (3.1 mM) in the presence of PbCl_2_ (Au nanorod: 0 µM, 0.04 µM, 0.08 µM, 0.2 µM, 0.3 µM, 0.8 µM, 1.7 µM, 5.6 µM and 11.2 µM, Au nanotriangle: 0 µM, 0.6 µM, 1.2 µM, 2.5 µM, 5.0 µM, 9.0 µM and 18.0 µM). TEM images of Au nanorods and Au nanotriangles are inset.

As demonstrated in Figure 5, it is revealed that Au nanorods exhibit lower LOD than Au nanotriangles [55,69]; it is noteworthy that log-scale in Pb^2+^ concentration was applied for linear fitting (see Note S5 in the Supplementary). LOD for Pb^2+^ ions with Au nanorods was measured to be 68 nM, whereas it was 380 nM for Au nanotriangles, in which three times signal-to-noise (S/N ratio) rule was applied to the determination of LOD. Relatively poor LODs were caused by large errors (3σ) in conjunction with poor linearity (R2 = 0.984 and 0.947). Nevertheless, better LOD with nanorods might be attributed to relatively sharp LSPR band and red-shift of the peak wavelength as Pb^2+^-associated etching proceeds; we measured absorption values at fixed wavelength (see Figure 2a,c). On the other hand, Au nanotriangles show much wider LSPR band, indicating their polydispersity even after the separation process. However, the Au nanotriangles show higher sensitivity in a certain range of Pb^2+^-concentrations (0.6 μM~18 μM) since UV-Vis spectra of the Au nanotriangles resulted in a steeper slope than the Au nanorods.

To improve the reliability and lower the LOD of the current scheme for detecting Pb^2+^ ions, some sources of experimental error were deliberated. Foremost, the inherent etching of thiosulfate without Pb^2+^ ions seems to deteriorate sensor performances. As shown in Figure S3b, a gradual decrease in peak intensity, albeit small, was observed on a minute-by-minute basis, for that reason we tried to tightly keep the time interval to 5 min before adding 2-ME, but deviations of more than a few seconds were inevitable. The second source is the polydispersity of Au nanorods and Au nanotriangles. As mentioned above, the sharper LSPR bands of the separated Au nanorods showed a lower LOD than that of the Au nanotriangles, but the TEM analyses shown in Figure 3d,e suggest that even the sharp LSPR band is composed of distinct Au nanorods with a certain range of dimensions. Therefore, a methodology enhancing monodispersity (in synthetic or separating protocols) could be directly linked to better performance for Pb^2+^ ion sensors. In fact, dark-field spectroscopic measurement for single Au nanoparticle demonstrated much lower LOD, up to 0.2 pM [70]. The last possible source of error might be the inconsistent preparation of Au nanorods and Au nanotriangles; the separation of as grown Au nanorods demonstrate quite good reproducibility but reproducing all the same LSPR bands in terms of peak wavelength, bandwidth, and peak intensity was almost impossible. Thus, in batch-to-batch comparisons, we attempted to carefully maintain the peak intensity of Au nanorods and Au nanotriangles in a tight range through dilution within a range of wavelengths (920~960 nm).

Considering the similar growth conditions (CTA^+^ micelles) of two nanomaterials, the main differences can be ascribed to different facets of the two Au nanostructures. As schematically shown in Figure 4g, Au nanorods grown from spherical seeds consist of pentahedral and twined {111}-faceted end caps and {100}-faceted sidewalls. On the other hand, Au nanotriangles grown in similar conditions were composed of Au{111}-faceted flat surfaces and {100}-faceted thin sidewalls. Therefore, Au{100} planes are the most abundantly exposed surfaces for Au nanorods whereas Au{111} planes for Au nanotriangles. Furthermore, Pb^2+^ ions have a strong tendency to form alloys with Au on the surface, obvious in the field of metallic surface sciences [71,72]. In addition, Pb adsorption and its thermodynamics are well-known and manifested in Pb underpotential deposition in the community of electrochemistry [66]. In a typical underpotential deposition on polycrystalline Au surfaces (via cyclic voltammetry), Pb atoms on Au{111} planes were stripped out first (in an oxidation sweep) and deposited last (in a reduction sweep), suggesting that Au{111} planes are the least favorable toward the alloy formation than other planes such as Au{100} and {110} [66].

From this background information, our experimental observations suggest that the two opposing spectroscopic evolutions of Au nanorods and Au nanotriangles might be related to the alloy formation of Pb on the gold surface. We envision that Pb^2+^ ions coexisting with 2-ME/Na_2_S_2_O_3_, predominantly form alloys on Au{100} surfaces, and subsequently accelerate the etching rate of Au{100} planes compared to Au{111} planes. This is quite plausible when reminding that Au{111} surfaces are the most densely packed surface planes considering the *fcc* structure of Au single crystals, as schematically shown in Figure S5. Moreover, this alloy formation elaborates the selectivity of Pb^2+^ ions over other heavy metal ions; it is assumed that only Pb^2+^ ions in solution phase can form Au-Pb alloy for the current etching conditions (Figure S6); selectivity for Pb^2+^ ions for Au nanomaterials was also reported in the literature [12,61,73,74]. In addition, it has been known in the field of metallurgy that the formation of AuPb_2_ crystals enhances leaching gold, where thiourea was used as an active ligand [75,76]. Thus, we reasoned that excessive adsorption (and alloy formation) of Pb^2+^ ions onto Au{100} planes compared to Au{111} and subsequent faster etching by 2-ME/S_2_O_3_^2−^ pair is a crucial step for increasing the aspect ratio of Au nanorods.

Acceleration of the etching rate by Pb^2+^ ions has been employed in the development of advanced Pb^2+^ optical sensors. For instance, Au nanostar (blue solution initially) become spherical Au nanoparticle at the end point (showing typical wine color of spherical nanoparticle) and demonstrated picomolar detection limit for Pb^2+^ ions [66], which was facilitated by high surface area of the Au nanostar. Interestingly, X-ray photoelectron spectroscopic (XPS) data could show the presence of elemental lead (Pb 4f_7/2_ and 4f_5/2_ bands), on which no obvious reductant for Pb^2+^ was designated. In a separate SALDI-TOF MS (surface-assisted laser desorption ionization-time of flight mass spectroscopy) study, nanoclusters composed of [Au_9-m_Pb_m_]^+^ (m: 0~8) were also reported, implicating the presence of Au-Pb alloy [61]. Their reaction condition (2-ME: ~1 mM, Na_2_S_2_O_3_: ~2 mM) was rather mild and very similar to ours, and maintained selectivity over other heavy metal ions. In more field-oriented applications, spherical Au nanoparticles were embedded in polymer matrix as a solid-phase Pb-detecting optical sensor, boasting much higher sensitivity toward Pb^2+^ ions, which is aided by co-operative dissolution of Au nanoparticles from polymeric matrix as Pb^2+^ concentration increases [12]. All those Pb^2+^ ion detection was performed in the presence of 2-ME/Na_2_S_2_O_3_ and assumed to involve the formation of Au-Pb alloy.

## 5. Conclusions

Ambiguous about the role of Pb^2+^ ions, we have started an investigation upon the Pb^2+^-assisted leaching phenomena of Au nanomaterials in the presence of 2-ME and S_2_O_3_^2−^ ions; Au nanorods and Au nanotriangles were selected as representative species. Indeed, Pb^2+^ ions are observed to be crucial for the acceleration of the etching rate for both Au nanomaterials, which is primarily powered by 2-ME and S_2_O_3_^2−^ pair. In UV-Vis spectroscopic investigation, Au nanorods showed much lower LOD than that of Au nanotriangles, mainly owing to better monodispersity (thus a sharper LSPR band in UV-Vis spectrum). Au nanorods, to our surprise, experienced an increase in aspect ratio while reducing their overall size at the etching stage, elaborating its red-shift in UV-Vis spectrum. On the other hand, Au nanotriangles surrounded by mostly dense Au{111} planes on two flat sides did not show the red-shift in UV-Vis spectrum, but showed a consistent blue-shift. We believe that facet-dependent alloy formation of Au with Pb^2+^ ions plays a central role in these unprecedented characteristics and might happen without any specific reductant for Pb^2+^ ions. These findings here on Au nanorods seems to have rich implications on the development of novel probes for Pb^2+^ ions that are embedded in biological tissues [77,78], since the deeper penetration depth of NIR is crucial for future transdermal sensors for Pb^2+^ ions [67].

## Data Availability

Not applicable.

## Supplementary Materials

The following supporting information can be downloaded at: https://www.mdpi.com/article/10.3390/s24020497/s1, Figure S1: UV-Vis absorption spectra of as-grown Au nanomaterials; Figure S2: Dot plot of UV-Vis absorbance values of Au nanorods at 961 nm for prolonged incubation time; Figure S3: Time course of UV-Vis spectra of Au nanorods on sequentially added etchant; Figure S4: Enlarged UV-Vis spectra in Figure S3 was retrieved; Figure S5: A schematic cartoon of Au surface while Au-Pb alloy is formed and etched in facet-dependent way; Figure S6: Selectivity test of the 2-ME/S_2_O_3_^2−^ etching system toward various metal ions; Table S1: The top table summarizes particle type, signal change, and experimental conditions while the bottom table shows the maximum peak position and FWHM of UV-Vis spectra; Table S2: The summary table for Pb^2+^ detection papers which are closely related with this study. Note S1: How were the syntheses of Au nanorods and Au nanotriangles performed in detail?; Note S2: Why haven’t used any stabilizer without etching properties, if the etching ability of thiosulfate has been expected?; Note S3: Does 2-ME have the capability to etch gold?; Note S4: How the LOD was determined?; Note S5: Are there any physical reasons for the relationship between the derived (A^0^−A)/A^0^) parameter and concentration?

## References

[1] WHO (2017). Guidelines for Drinking-Water Quality.

[2] (2009). U.S. EPA National Primary Drinking Water Regulations (EPA 816-F-09-004).

[3] Needleman H. (2004). Lead Poisoning. Annu. Rev. Med..

[4] Järup L. (2003). Hazards of heavy metal contamination. Br. Med. Bull..

[5] Ghaedi M., Shokrollahi A., Niknam K., Niknam E., Najibi A., Soylak M. (2009). Cloud point extraction and flame atomic absorption spectrometric determination of cadmium(II), lead(II), palladium(II) and silver(I) in environmental samples. J. Hazard. Mater..

[6] Afonso D.D., Baytak S., Arslan Z. (2010). Simultaneous generation of hydrides of bismuth, lead and tin in the presence of ferricyanide and application to determination in biominerals by ICP-AES. J. Anal. At. Spectrom..

[7] Michalska A., Wojciechowski M., Wagner B., Bulska E., Maksymiuk K. (2006). Laser Ablation Inductively Coupled Plasma Mass Spectrometry Assisted Insight into Ion-Selective Membranes. Anal. Chem..

[8] Deibler K., Basu P. (2013). Continuing Issues with Lead: Recent Advances in Detection. Eur. J. Inorg. Chem..

[9] Vantelon D., Lanzirotti A., Scheinost A.C., Kretzschmar R. (2005). Spatial Distribution and Speciation of Lead around Corroding Bullets in a Shooting Range Soil Studied by Micro-X-ray Fluorescence and Absorption Spectroscopy. Environ. Sci. Technol..

[10] Zhang R., Li L., Sultanbawa Y., Xu Z.P. (2018). X-ray fluorescence imaging of metals and metalloids in biological systems. Am. J. Nucl. Med. Mol. Imaging.

[11] Wei Y., Yang R., Yu X.-Y., Wang L., Liu J.-H., Huang X.-J. (2012). Stripping voltammetry study of ultra-trace toxic metal ions on highly selectively adsorptive porous magnesium oxide nanoflowers. Analyst.

[12] Ferhan A.R., Guo L., Zhou X., Chen P., Hong S., Kim D.-H. (2013). Solid-Phase Colorimetric Sensor Based on Gold Nanoparticle-Loaded Polymer Brushes: Lead Detection as a Case Study. Anal. Chem..

[13] Shrivas K., Sahu B., Deb M.K., Thakur S.S., Sahu S., Kurrey R., Kant T., Patle T.K., Jangde R. (2019). Colorimetric and paper-based detection of lead using PVA capped silver nanoparticles: Experimental and theoretical approach. Microchem. J..

[14] Singh H., Bamrah A., Bhardwaj S.K., Deep A., Khatri M., Brown R.J.C., Bhardwaj N., Kim K.-H. (2021). Recent advances in the application of noble metal nanoparticles in colorimetric sensors for lead ions. Environ. Sci. Nano.

[15] Nguyen H., Sung Y., O’Shaughnessy K., Shan X., Shih W.C. (2018). Smartphone Nanocolorimetry for On-Demand Lead Detection and Quantitation in Drinking Water. Anal. Chem..

[16] Fakhri N., Hosseini M., Tavakoli O. (2018). Aptamer-based colorimetric determination of Pb2+ using a paper-based microfluidic platform. Anal. Methods.

[17] Sahu B., Kurrey R., Deb M.K., Shrivas K., Karbhal I., Khalkho B.R. (2021). A simple and cost-effective paper-based and colorimetric dual-mode detection of arsenic(iii) and lead(ii) based on glucose-functionalized gold nanoparticles. RSC Adv..

[18] Tian W., Wang D., Fan H., Yang L., Ma G. (2018). A Plasma Biochemical Analysis of Acute Lead Poisoning in a Rat Model by Chemometrics-Based Fourier Transform Infrared Spectroscopy: An Exploratory Study. Front. Chem..

[19] Lehmann S., Fischer M., Rosin A., Gerdes T., Krenkel W. (2020). The feasibility of CO_2_-laser-induced breakdown spectroscopy for fast lead determination in glass cullet. Int. J. Appl. Glass Sci..

[20] Song R., Zhang Q., Chu Y., Zhang L., Dai H., Wu W. (2019). Fluorescent cellulose nanocrystals for the detection of lead ions in complete aqueous solution. Cellulose.

[21] Chen H., Shao S., Yu Y., Huang Y., Zhu X., Zhang S., Fan J., Yin G.Y., Chi B., Wan M. (2020). A dual-responsive biosensor for blood lead detection. Anal. Chim. Acta.

[22] Silva I.B., de Araújo D.M., Vocciante M., Ferro S., Martínez-Huitle C.A., Dos Santos E.V. (2021). Electrochemical Determination of Lead Using A Composite Sensor Obtained from Low-Cost Green Materials: Graphite/Cork. Appl. Sci..

[23] Kim H.N., Ren W.X., Kim J.S., Yoon J. (2012). Fluorescent and colorimetric sensors for detection of lead, cadmium, and mercury ions. Chem. Soc. Rev..

[24] Chae M.-Y., Yoon J., Czarnik A.W. (1996). Chelation-enhanced fluorescence chemosensing of Pb(II), an inherently quenching metal ion. J. Mol. Recognit..

[25] Meng X., Cao D., Hu Z., Han X., Li Z., Ma W. (2018). A highly sensitive and selective chemosensor for Pb^2+^ based on quinoline–coumarin. RSC Adv..

[26] Singh R., Das G. (2019). “Turn-on” Pb^2+^ sensing and rapid detection of biothiols in aqueous medium and real samples. Analyst.

[27] Qi D., Zhang J., Zhang D., Zhu M., Gong L., Su C., Lu W., Bian Y., Jiang J. (2020). A phthalocyanine-porphyrin triad for ratiometric fluorescent detection of Lead(II) ions. Dye. Pigment..

[28] Métivier R., Leray I., Valeur B. (2003). A highly sensitive and selective fluorescent molecular sensor for Pb(ii) based on a calix[4]arene bearing four dansyl groups. Chem. Commun..

[29] Liu J.-M., Bu J.-H., Zheng Q.-Y., Chen C.-F., Huang Z.-T. (2006). Highly selective fluorescent sensing of Pb^2+^ by a new calix[4]arene derivative. Tetrahedron Lett..

[30] Buie N.M., Talanov V.S., Butcher R.J., Talanova G.G. (2008). New Fluorogenic Dansyl-Containing Calix[4]arene in the Partial Cone Conformation for Highly Sensitive and Selective Recognition of Lead(II). Inorg. Chem..

[31] Kim I.-B., Dunkhorst A., Gilbert J., Bunz U.H.F. (2005). Sensing of Lead Ions by a Carboxylate-Substituted PPE:  Multivalency Effects. Macromolecules.

[32] Narkwiboonwong P., Tumcharern G., Potisatityuenyong A., Wacharasindhu S., Sukwattanasinitt M. (2011). Aqueous sols of oligo(ethylene glycol) surface decorated polydiacetylene vesicles for colorimetric detection of Pb^2+^. Talanta.

[33] Li J., Lu Y. (2000). A Highly Sensitive and Selective Catalytic DNA Biosensor for Lead Ions. J. Am. Chem. Soc..

[34] Swearingen C.B., Wernette D.P., Cropek D.M., Lu Y., Sweedler J.V., Bohn P.W. (2005). Immobilization of a Catalytic DNA Molecular Beacon on Au for Pb(II) Detection. Anal. Chem..

[35] Wernette D.P., Swearingen C.B., Cropek D.M., Lu Y., Sweedler J.V., Bohn P.W. (2006). Incorporation of a DNAzyme into Au-coated nanocapillary array membranes with an internal standard for Pb(ii) sensing. Analyst.

[36] Guo Y., Li J., Zhang X., Tang Y. (2015). A sensitive biosensor with a DNAzyme for lead(ii) detection based on fluorescence turn-on. Analyst.

[37] Wang H.-B., Ma L.-H., Fang B.-Y., Zhao Y.-D., Hu X.-B. (2018). Graphene oxide-assisted Au nanoparticle strip biosensor based on GR-5 DNAzyme for rapid lead ion detection. Colloids Surf. B Biointerfaces.

[38] Kim Y., Johnson R.C., Hupp J.T. (2001). Gold Nanoparticle-Based Sensing of “Spectroscopically Silent” Heavy Metal Ions. Nano Lett..

[39] Berlina A.N., Zherdev A.V., Pridvorova S.M., Gaur M.S., Dzantiev B.B. (2019). Rapid Visual Detection of Lead and Mercury via Enhanced Crosslinking Aggregation of Aptamer-Labeled Gold Nanoparticles. J. Nanosci. Nanotechnol..

[40] Dehghani Z., Hosseini M., Mohammadnejad J., Ganjali M.R. (2019). Novel colorimetric sensor based on peroxidase-like activity of chitosan-stabilized Au/Pt nanoclusters for trace lead. Anal. Methods.

[41] Solra M., Bala R., Wangoo N., Soni G.K., Kumar M., Sharma R.K. (2020). Optical pico-biosensing of lead using plasmonic gold nanoparticles and a cationic peptide-based aptasensor. Chem. Commun..

[42] Sahu S., Sharma S., Ghosh K.K. (2020). Novel formation of Au/Ag bimetallic nanoparticles from a mixture of monometallic nanoparticles and their application for the rapid detection of lead in onion samples. New J. Chem..

[43] Liu D.-M., Dong C. (2023). Gold nanoparticles as colorimetric probes in food analysis: Progress and challenges. Food Chem..

[44] Yu L., Song Z., Peng J., Yang M., Zhi H., He H. (2020). Progress of gold nanomaterials for colorimetric sensing based on different strategies. TrAC Trends Anal. Chem..

[45] Zhu D., Zhang X., Han Y., Luan X., Wei G. (2023). Biomimetic gold nanomaterials for biosensing, bioimaging and biotherapy: A mini-review. Sens. Diagn..

[46] Sanchis-Gual R., Coronado-Puchau M., Mallah T., Coronado E. (2023). Hybrid nanostructures based on gold nanoparticles and functional coordination polymers: Chemistry, physics and applications in biomedicine, catalysis and magnetism. Coord. Chem. Rev..

[47] Liu S., Min X., Xiang M., Wang J., Tang L., Liu L. (2022). Nanoanalysis of the leaching process simulation of Pb in agricultural soil. Environ. Pollut..

[48] Hua Z., Yu T., Liu D., Xianyu Y. (2021). Recent advances in gold nanoparticles-based biosensors for food safety detection. Biosens. Bioelectron..

[49] Gad G.M.A., Hegazy M.A. (2019). Optoelectronic properties of gold nanoparticles synthesized by using wet chemical method. Mater. Res. Express.

[50] Chen J., Gan F. (2022). Polarization-switchable plasmonic emitters based on laser-induced bubbles. Opto-Electron. Adv..

[51] Jing J., Liu K., Jiang J., Xu T., Wang S., Liu T. (2023). Highly sensitive and stable probe refractometer based on configurable plasmonic resonance with nano-modified fiber core. Opto-Electron. Adv..

[52] Yuan Z., Hu C.-C., Chang H.-T., Lu C. (2016). Gold nanoparticles as sensitive optical probes. Analyst.

[53] Li Y., Lin H., Zhou W., Sun L., Samanta D., Mirkin C.A. (2021). Corner-, edge-, and facet-controlled growth of nanocrystals. Sci. Adv..

[54] Rao H., Xue X., Wang H., Xue Z. (2019). Gold nanorod etching-based multicolorimetric sensors: Strategies and applications. J. Mater. Chem. C.

[55] Zhang Y., Leng Y., Miao L., Xin J., Wu A. (2013). The colorimetric detection of Pb^2+^ by using sodium thiosulfate and hexadecyl trimethyl ammonium bromide modified gold nanoparticles. Dalton Trans..

[56] Zhu J., Yu Y.-Q., Li J.-J., Zhao J.-W. (2016). Colorimetric detection of lead(ii) ions based on accelerating surface etching of gold nanorods to nanospheres: The effect of sodium thiosulfate. RSC Adv..

[57] Aylmore M.G., Muir D.M. (2001). Thiosulfate leaching of gold—A review. Miner. Eng..

[58] Ke C.-Y., Chen T.-H., Lu L.-C., Tseng W.-L. (2014). Understanding thiol-induced etching of luminescent gold nanoclusters. RSC Adv..

[59] Ha T.H., Koo H.-J., Chung B.H. (2007). Shape-Controlled Syntheses of Gold Nanoprisms and Nanorods Influenced by Specific Adsorption of Halide Ions. J. Phys. Chem. C.

[60] Smith D.K., Korgel B.A. (2008). The Importance of the CTAB Surfactant on the Colloidal Seed-Mediated Synthesis of Gold Nanorods. Langmuir.

[61] Chen Y.-Y., Chang H.-T., Shiang Y.-C., Hung Y.-L., Chiang C.-K., Huang C.-C. (2009). Colorimetric Assay for Lead Ions Based on the Leaching of Gold Nanoparticles. Anal. Chem..

[62] Link S., Mohamed M.B., El-Sayed M.A. (1999). Simulation of the Optical Absorption Spectra of Gold Nanorods as a Function of Their Aspect Ratio and the Effect of the Medium Dielectric Constant. J. Phys. Chem. B.

[63] Lan Y.-J., Lin Y.-W. (2014). A non-aggregation colorimetric method for trace lead(ii) ions based on the leaching of gold nanorods. Anal. Methods.

[64] Abbruzzese C., Fornari P., Massidda R., Vegliò F., Ubaldini S. (1995). Thiosulphate leaching for gold hydrometallurgy. Hydrometallurgy.

[65] Dreier T.A., Ackerson C.J. (2015). Radicals Are Required for Thiol Etching of Gold Particles. Angew. Chem. Int. Ed..

[66] Mayet N., Servat K., Kokoh K.B., Napporn T.W. (2019). Probing the Surface of Noble Metals Electrochemically by Underpotential Deposition of Transition Metals. Surfaces.

[67] Lane L.A., Xue R., Nie S. (2018). Emergence of two near-infrared windows for in vivo and intraoperative SERS. Curr. Opin. Chem. Biol..

[68] Wang S., Huang X., An Q., Zhou R., Xu W., Xu D., Lin Q., Cao X. (2021). Gold nanostar as an ultrasensitive colorimetric probe for picomolar detection of lead ion. Anal. Chim. Acta.

[69] Nguyen N.L.T., Kim E.J., Chang S.-K., Park T.J. (2016). Sensitive detection of lead ions using sodium thiosulfate and surfactant-capped gold nanoparticles. BioChip J..

[70] Dai D., Xu D., Cheng X., He Y. (2014). Direct imaging of single gold nanoparticle etching: Sensitive detection of lead ions. Anal. Methods.

[71] Biberian J.P., Rhead G.E. (1973). Spontaneous alloying of a gold substrate with lead monolayers. J. Phys. F Metal Phys..

[72] Perdereau J., Biberian J.P., Rhead G.E. (1974). Adsorption and surface alloying of lead monolayers on (111) and (110) faces of gold. J. Phys. F Metal. Phys..

[73] Lee Y.-F., Huang C.-C. (2011). Colorimetric Assay of Lead Ions in Biological Samples Using a Nanogold-Based Membrane. ACS Appl. Mater. Interfaces.

[74] Lee Y.-F., Nan F.-H., Chen M.-J., Wu H.-Y., Ho C.-W., Chen Y.-Y., Huang C.-C. (2012). Detection and removal of mercury and lead ions by using gold nanoparticle-based gel membrane. Anal. Methods.

[75] Soleymani M., Li L., Sadri F., Ghahreman A. (2022). The electrochemical catalytic role of Pb^2+^ in thiosulfate gold oxidation process. Miner. Eng..

[76] Li K., Zhang Y., Li Q., Liu X., Yang Y., Jiang T. (2023). Role of foreign ions in the thiourea leaching of gold. Miner. Eng..

[77] Xiao Z., Ji C., Shi J., Pridgen E.M., Frieder J., Wu J., Farokhzad O.C. (2012). DNA Self-Assembly of Targeted Near-Infrared-Responsive Gold Nanoparticles for Cancer Thermo-Chemotherapy. Angew. Chem..

[78] Li N., Zhao P., Astruc D. (2014). Anisotropic Gold Nanoparticles: Synthesis, Properties, Applications, and Toxicity. Angew. Chem. Int. Ed..

