# Supplementary material for: Pb2+ Ion Sensors Employing Gold Etching Process: Comparative Investigation on Au Nanorods and Au Nanotriangles
Source: Sensors (Basel). 2024 Jan 13;24(2):497. doi: 10.3390/s24020497 (PMC10820728; doi:10.3390/s24020497)
Supplement: Supplementary file 1 [file sensors-24-00497-s001.zip › sensors-2791310-supplementary.pdf]

Supporting information

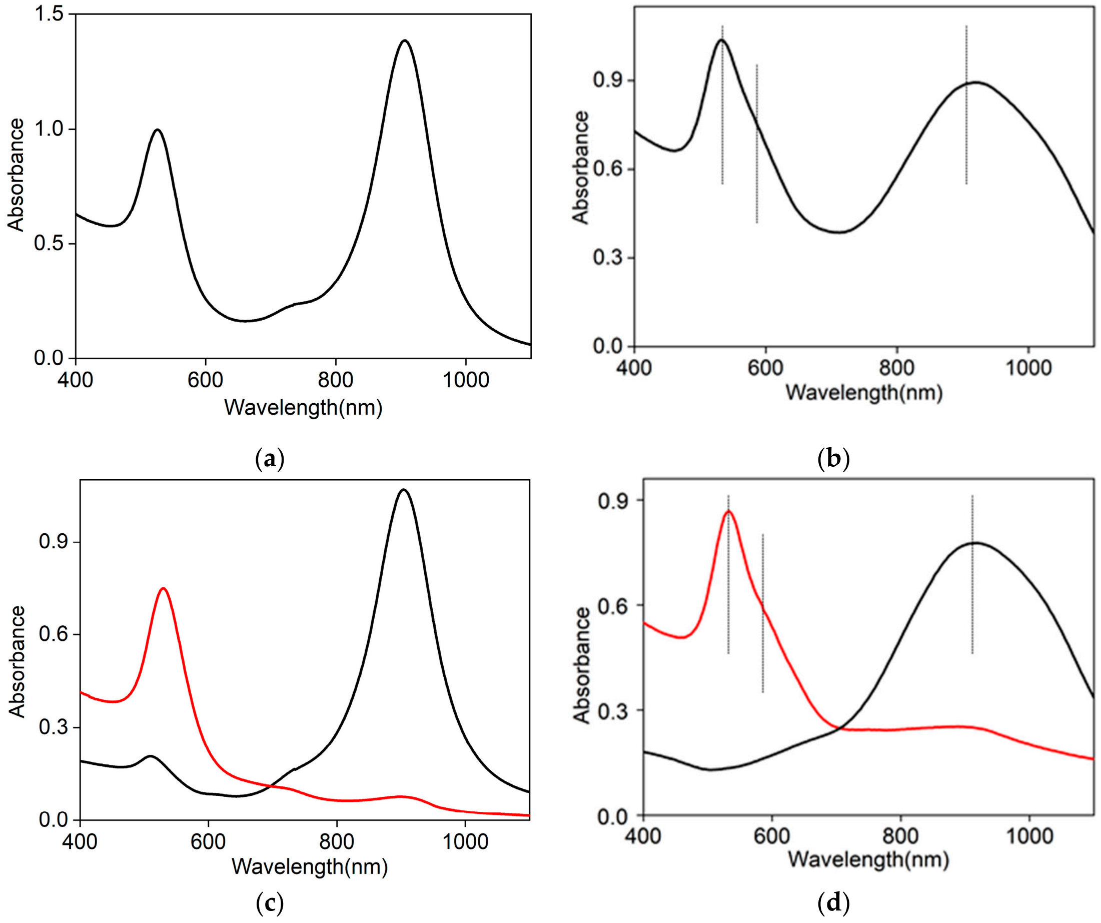

Figure S1. UV-Vis absorbance spectra of (a) as-grown Au nanorods and (b) as-grown Au nanotriangles (c) after salting out Au nanorods and (d) after salting out Au nanotriangles. ((c), (d) red: supernatant, black: resuspended precipitate)

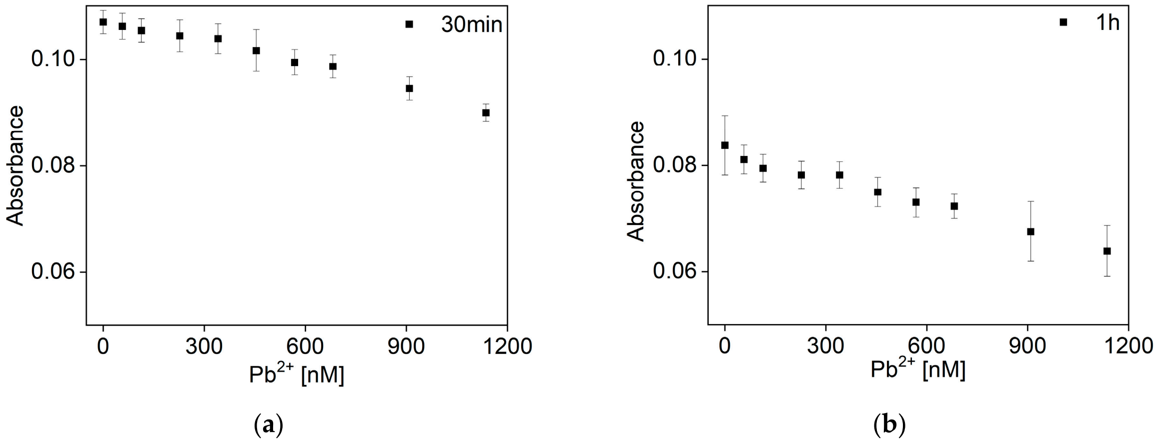

Figure S2. Dot plot of UV-Vis absorbance values at 961 nm of Au nanorods reacted with $Na_2S_2O_3$ (0.9 mM), 2-ME (4.1 mM) and various concentrations of Pb^{2+} (a) after 30 min incubation and (b) 1 h incubation.

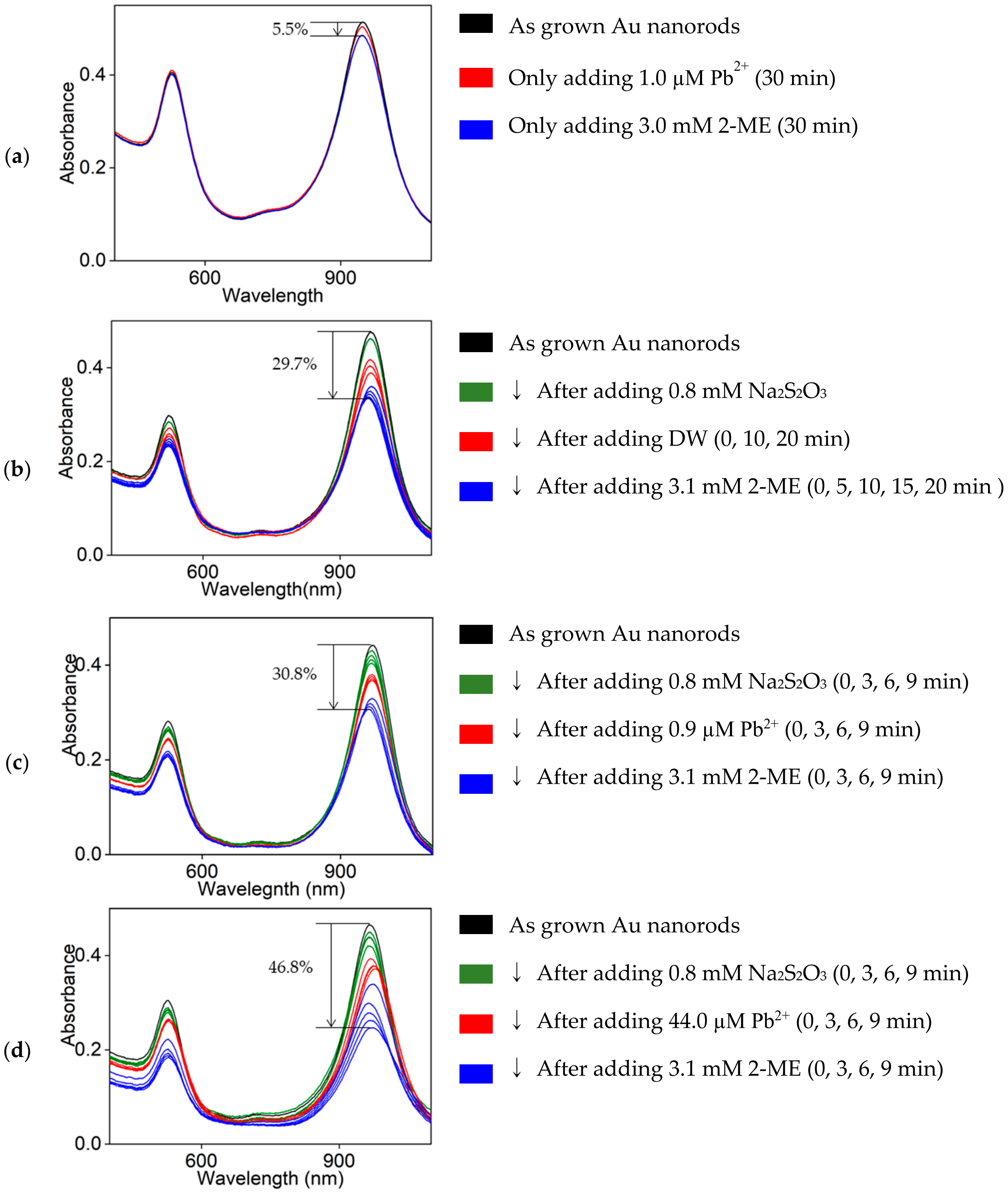

Figure S3. UV-Vis absorbance spectra of Au nanorods with time interval. (a) As grown Au nanorods (black), Au nanorods with 1.0 µM Pb^{2+}(red), and Au nanorods with 3.0 mM 2-ME (blue). (b) Au nanorods after sequentially adding 0.8 mM $Na_2S_2O_3$, 0 µM $PbCl_2$ and 3.1 mM 2-ME with time interval of 10 min. (c) Au nanorods after sequentially adding 0.8 mM $Na_2S_2O_3$, 0.9 µM $PbCl_2$ and 3.1 mM 2-ME with time interval of 3 min. (d) Au nanorods after sequentially adding 0.8 mM $Na_2S_2O_3$, 44.0 µM $PbCl_2$ and 3.1 mM 2-ME with time interval of 3 min.

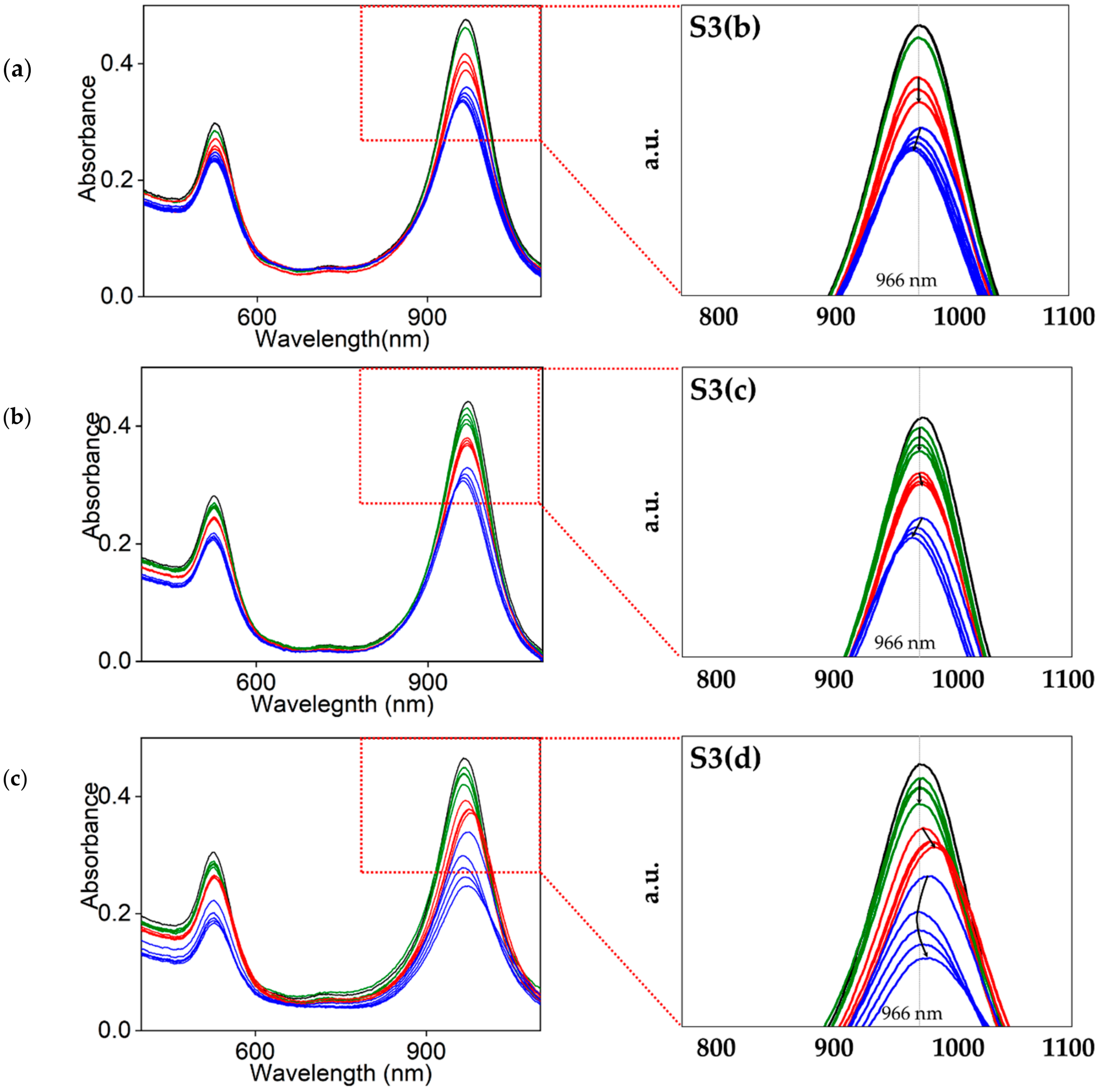

Figure S4. Enlarged UV-Vis spectra in Figure S3 was retrieved.

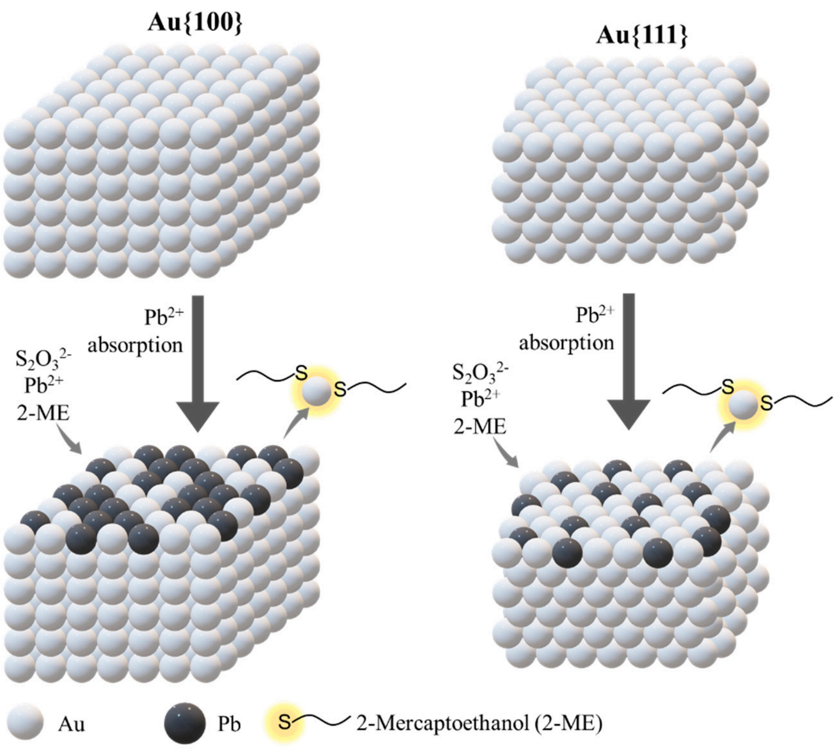

Figure S5. A schematic cartoon of Au surface while Au-Pb alloy is formed and etched in facet-dependent way.

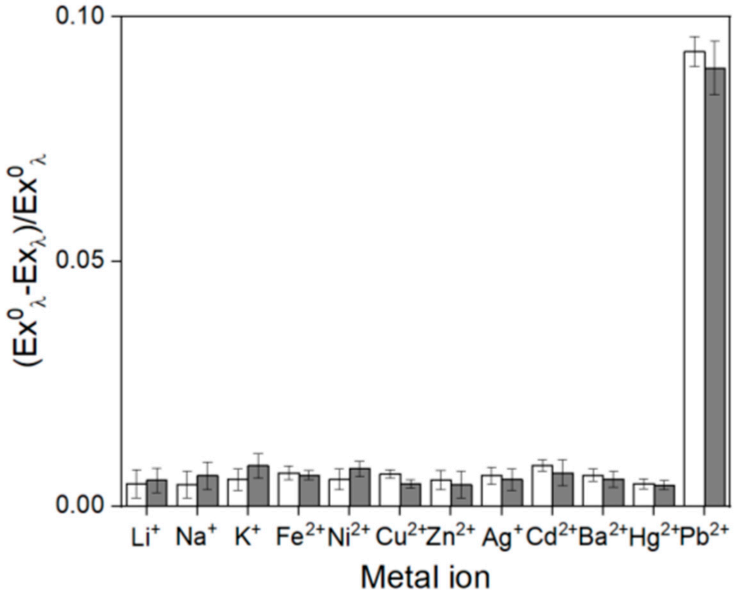

Figure S6. Selectivity test of the 2-ME/$S_2O_3^{2-}$ etching system toward various metal ions. The concentration of metal ions was 5 μM except Pb^{2+} ion (100 nM). Error bars represent two times of standard deviations from three repeated experiments. Other conditions were the same as those described in the experimental section. White bar is conducted with Au nanorods while gray bar with Au nanotriangles.

(a)

Figure 1(b) in main text	
Particle type	Au nanorod
Signal change	Red shift.<br>Absorbance 15.0% decrease
Experimental condition	Time 30min<br>$[Pb^{2+}]$ 0~12 μM<br>$[S_2O_3^{2-}]$ 0.8 mM<br>[2-ME] 3.0 mM

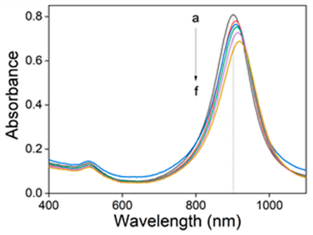

	a. 0 μM	b. 1 μM	c. 2 μM	d. 4 μM	e. 8 μM	f. 12 μM
Maximum peak (nm)	902	909	909	911	915	918
FWHM	121	123	130	124	126	130

(b)

Figure 2(a) in main text	
Particle type	Au nanorod
Signal change	Red shift.<br>Absorbance 30.7% decrease
Experimental condition	Time 30min<br>$[Pb^{2+}]$ 0~111.2 μM<br>$[S_2O_3^{2-}]$ 0.8 mM<br>[2-ME] 3.1 mM

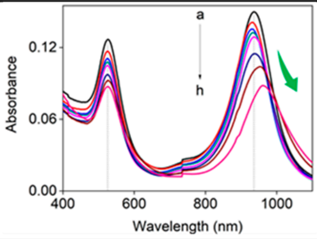

	a. 0 μM	b. 0.3 μM	c. 0.8 μM	d. 1.7 μM	e. 5.6 μM	g. 27.6 μM	h. 55.6 μM	i. 111.2 μM
Maximum peak (nm)	936	931	930	932	935	938	953	961
FWHM	126	127	126	125	130	136	155	160

(c)

Figure 2(c) in main text	
Particle type	Au nanorod
Signal change	Red shift.<br>Absorbance 30.1% decrease
Experimental condition	Time 30min<br>$[Pb^{2+}]$ 0~1.10 μM<br>$[S_2O_3^{2-}]$ 0.2 mM<br>[2-ME] 22.1 mM

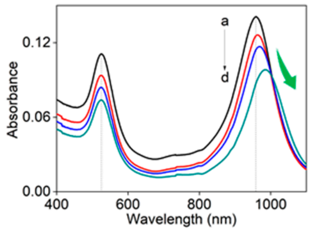

	a. 0 μM	b. 0.11 μM	c. 33 μM	d. 1.1 μM
Maximum peak (nm)	959	963	968	985
FWHM	132	127	131	136

(d)

Figure 4(a) in main text	
Particle type	Au nanotriangle
Signal change	Blue shift.<br>Absorbance 47.9% decrease
Experimental condition	Time 30min<br>$[Pb^{2+}]$ 0~72.0 μM<br>$[S_2O_3^{2-}]$ 1.0 mM<br>[2-ME] 3.8 mM

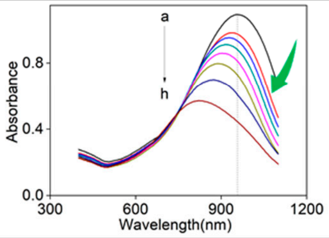

	a. 0 μM	b. 2.5 μM	c. 5 μM	d. 9 μM	e. 18 μM	f. 27 μM	g. 54 μM	h. 72 μM
Maximum peak (nm)	957	931	931	919	905	888	871	821
FWHM	-	348	345	341	336	338	377	407

* FWHM: Full Width at Half Maximum

Table S1. The top table summarizes particle type, signal change, and experimental conditions while the bottom table shows the maximum peak position and FWHM of UV-Vis spectra for each figure, (a) Figure 1(b), (b) Figure 2(a), (c) Figure 2(c), (d) Figure 4(a) in the main text.

No.	Technique	Etching agent	$[S_2O_3^{2-}]/[2\text{-}ME]$	Particle type	Surfactant	Wavelength	LOD	Time	Comment	Ref.
1	Colorimetry	$S_2O_3^{2-}$/2-ME	1 mM/1 mM	Nanosphere	citrate	Red shift (Aggregation)	0.5 nM	1.5 h		[61]
3	Colorimetry	$S_2O_3^{2-}$/2-ME	0.1 M/250mM	Nanosphere	BSA	-	45 nM	< 10 min		[73]
4	Colorimetry	$S_2O_3^{2-}$/2-ME	1 mM/0.1 M	Nanosphere	BSA	-	50 pM	1.5 h		[74]
5	Colorimetry	$S_2O_3^{2-}$	6.7 mM	Nanosphere	CTAB	-	40 nM	30 min		[55]
6	Colorimetry	$S_2O_3^{2-}$/2-ME	1 mM/1 mM	Nanosphere	POEGMA*	-	25 pM	45 min	Polymer brush matrix	[12]
7	Microscopy	$S_2O_3^{2-}$/2-ME	0.05 mM/1 mM	Nanosphere	citrate	-	0.2 pM	6 h	Single particle detection	[69]
8	Colorimetry	$S_2O_3^{2-}$	3.0 mM	Nanorod	CTAB	-	4.3 nM	30 min		[63]
9	Colorimetry	$S_2O_3^{2-}$	3.6 mM	Nanorod	CTAB	Blue shift	20 nM	5 min		[56]
10	Colorimetry	$S_2O_3^{2-}$/2-ME	0.8 mM/3 mM	Nanorod	CTAC	Red shift	73 nM	30 min		This study
11	Colorimetry	$S_2O_3^{2-}$/2-ME	1 mM/3.8 mM	Nanotriangle	CTAB	Blue shift	380 nM	30 min		This study

* POEGMA: poly(oligo(ethylene glycol)methacrylate)

Table S2. The summary table for Pb^{2+} detection papers which are closely related with this study.

The following notes provide supplementary explanations in Q&A format to help readers understand issues that may arise while reading the text. Those issues are denoted in the main text, for instance, as "see note 1".

Note S1:
Q: How were the syntheses of Au nanorods and Au nanotriangles performed in detail?

A: First of all, sodium borohydride is a strong reducing agent that often adopted to make Au seeds (3 ~ 4 nm) for 'seeded growth of Au nanorods'. Borohydride is so powerful that almost autonomous seed formation is guaranteed, which is a critical step for general crystal growth concept for Au or Ag nanocrystals. Citrate ion is also commonly employed for passivating agent that prevent uncontrolled aggregation of as synthesized Au seeds, but it let further growth of Au nanocrystals in rather mild growth condition such as ascorbic acid as a reducing agent. The 'seeded growth of Au nanocrystals' has been a popular avenue for manufacturing of various Au nanomaterials, and excellent review articles can be found with names of prominent researchers (Luis M. Liz-marzán or Catherin J. Murphy)

The discoloration of growth solution by adding ascorbic acid is partial reduction of Au(III) into Au(I), which is commonly employed in seeded growth of Au nanocrystals. In ref.57, which is a result of our lab, we had found that a miniscule amount of iodide ion in the growth solution of concentrated CTAB produced Au nanotriangles, whereas, in its absence, produced linear Au nanorods as a major product; a certain range of bromide ions (sub-mM to hundred mM) were needed for Au nanorods. Right after our finding, many other groups could confirm that iodide ion in growth solution drove the Au nanoparticle growth dominantly into Au nanotriangles (with small amount of hexagonal nanoplates): L. M. Liz-Marzan et al. *ACS Nano* 2014, 8 (6), 5833-5842; C. J. Murphy et al. *Chem. Mater.* 2014, 26, 34-43. While iodide ion (10 ~ 80 μM) is essential for the formation of nanotriangle, the range of bromide concentration for nanorods synthesis is rather wide (500 μM ~ 100 mM) to a given CTAC solution (~0.07 M). As briefly mentioned in the original paper, Au nanorods could be formed even in CTAB solution (~0.07 M) solely, but with substantial amount of nanoplates (triangular or hexagonal) as by-products. Thus, addition of bromide ions to CTAC solution as major surfactant have been our choice for the growth of nanorods, which minimizes the growth of nanoplates.

Note S2:
Q: Why haven't used any stabilizer without etching properties, if the etching ability of thiosulfate has been expected?

A: The current reaction conditions to see the Pb^{2+}-assisted etching process here is based on the study of Huang et al. (ref.61). Among the wider range of experimental conditions in ref. 61, relatively mild condition was chosen to discriminate the facet-dependent etching in Au nanorods or Au nanotriangles (like longer incubation with low concentrations of thiosulfate and 2-ME). If we adopt stronger stabilizer (if any) like long PEG-thiol or other water-soluble thiol compounds, the Pb^{2+}-assisted etching would be almost quenched at lower concentration (~ nM) of Pb^{2+} and gold etching might not be observed. Otherwise, different results might be seen with introducing new components. In addition, finding a relevant stabilizer (or surfactant) would be another tedious study that can change the whole physicochemical environment of the etching solution, thereby disorienting the whole story. Hence, we'd like to maintain the current experimental situations without commenting upon another stabilizer.

Note S3:
Q: Does 2-ME have the capability to etch gold?

A: We have observed that 2-ME solely did not change the spectrum after initial drop of peak intensity, which can be largely attributed to 2-ME adsorption; but further evolution of UV-Vis spectrum was not recognized. Nevertheless, we'd like to note a report that 2-ME with radicals can etch gold surface (ref. 65), which, we assert, should be considered in reproducing or adopting this scheme for a variety of reasons. Also, when exposed to

high concentration of 2-ME or for long period of time, Au nanoparticles showed noticeably different (or degraded) features. In this study, 2-ME solely seems to be ineffective in terms of etching gold.

Note S4:
Q: How the LOD was determined?

A: According to LOD determining protocol, we have to first define 3σ noise level of blank sample (Pb^{2+} = 0 μM) or of overall experiments. Then, LOD was determined as x-values with the y-value of 3σ.

Note S5:
Q: Are there any physical reasons for the relationship between the derived ($(A^0$-$A)/A^0$) parameter and concentration?

A: The term of ($(A^0$-$A)/A^0$) is a normalized absorbance value. In lower concentrations of Pb^{2+} ions, absorbance intensity changes are more prominent compared to spectra in high concentration of Pb^{2+} ion that accompanies wavelength shift. As depicted in below, when we recorded the results (Figure 5) in linear scale, the Pb^{2+}-assisted etching shows a Pb^{2+} dependence that is definitely non-linear, indicating that the linear fitting failed to cover the whole concentration range. On the other hand, the *log*-scale plotting as in Fig 5 (absorbance changes versus *log*-scale Pb^{2+} concentration) that was adopted from ref. 61 (Huang et al.) can cover a much wider range of concentrations. This fitting resembles a Temkin isotherm (R.D. Johnson, F. H. Arnold, *Biochim. Biophys. Acta.* 1995 1247(2): 293-297; Saba A. Obaid *J. Phys.: Conf. Ser.* 2020, 1664 012011); in this model, adsorbing energy of a molecule decreases as the coverage on a surface increase. This fitting result insinuate a relation with Pb^{2+} adsorption onto Au surface, but it is unclear what the absorbance (y-axis) means in this heterogeneous samples; the current situation seems more complex than the simple Temkin model. In this line, even mentioning Temkin adsorption model, at this moment, seems misleading or quite premature without suggesting proper models and following reasoning. Instead, we simply noted that *log*-scale plotting is better for demonstrating the data or acquiring LOD values, and in-detail elaboration be left for future study.

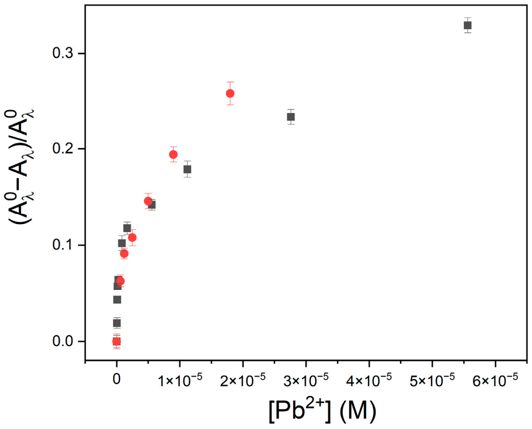

Linear scale plotting of Figure 5 in the main text (Black: Au nanorods, Red: Au nanotriangles).
